# Synthesis of *trans*-Mono(silyl)palladium(II) Bromide Complexes

**DOI:** 10.3390/molecules26092460

**Published:** 2021-04-23

**Authors:** Melvyn B. Ansell, George E. Kostakis, Oscar Navarro, John Spencer

**Affiliations:** 1 Department of Chemistry, School of Life Sciences, University of Sussex, Brighton BN1 9QJ, UK; mbansell25@gmail.com (M.B.A.); G.Kostakis@sussex.ac.uk (G.E.K.); 2 AMPAC Fine Chemicals, Highway 50 and Hazel Avenue, Building 05-019, Rancho Cordova, CA 95741-1718, USA

**Keywords:** palladium, silicon, N-heterocyclic carbene, allylsilane

## Abstract

The stoichiometric reaction of *cis*-[Pd(ITMe)_2_(SiR_3_)_2_], where (SiR_3_ = SiMe_3_ and SiMe_2_Ph and ITMe = 1,3,4,5-tetramethylimidazol-2-ylidene) with allyl bromide affords the corresponding allylsilanes along with complexes of the type *trans*-[Pd(ITMe)_2_(SiR_3_)(Br)]. The structure of *trans*-[Pd(ITMe)_2_(SiMe_2_Ph)Br] **2b** has been determined in the solid state and displays a slightly distorted square-planar geometry with the two N-heterocyclic carbene ligands in a *trans*-configuration.

## 1. Introduction

Mono(silyl)palladium(II) halide species are purported intermediates in a number of catalytic routes towards allylsilanes [1,2]. Palladium pincer chemistry accounts of such complexes are rather numerous, although examples of their isolation in this catalytic cycle are rare [3,4,5,6,7]. *Trans*-[PdCl(SiF_2_Ph)(L)_2_] (L = PMe_3_, PMe_2_Ph or PMePh_2_) and allyl bromide were shown to react to afford *trans*-[Pd(L)_2_(SiF_2_Ph)(Br)] and the corresponding allylsilane [1], and [(*^t^*BuPAr_2_)Pd(SiMe_3_)(I)] (Ar = 3,5-Me_2_-4-OMe-C_6_H_2_) was synthesized from stoichiometric quantities of [(cod)Pd(CH_2_SiMe_3_)_2_] (cod = 1,5-cyclooctadiene), *^t^*BuPAr_2_ and Me_3_SiI [2]. Analogues have been used in silyl-Negishi couplings [8]. We wish to report here our preliminary findings on the reaction of (ITMe)_2_Pd(silyl)_2_ complexes with allyl bromide (ITMe = 1,3,4,5-tetramethylimidazol-2-ylidene) [9,10,11,12].

## 2. Results and Discussion

**Scheme 1:**
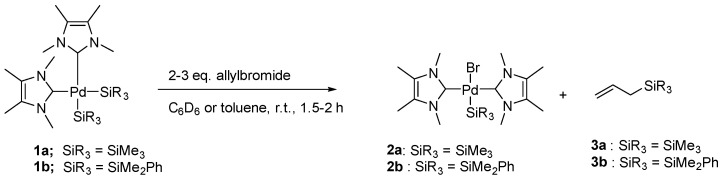
Stoichiometric synthesis of mono(silyl)palladium bromide complexes.

The bis(silyl)palladium complexes, *cis*-[Pd(ITMe)_2_(SiR_3_)_2_] (**1a**: SiR_3_ = SiMe_3_ and **1b**: SiMe_2_Ph [13,14] were reacted with excess allylbromide at room temperature under an nitrogen atmosphere to yield *trans*-[Pd(ITMe)_2_(SiMe_3_)(Br)] **2a** and *trans*-[Pd(ITMe)_2_(SiMe_2_Ph)Br] **2b** in 92 and 93% yield, respectively (Scheme 1). Reaction progress was monitored by ^1^H NMR spectroscopy. Characteristic resonances corresponding to silanes **3a** and **3b** were observed (in a 1:1 stoichiometry with **2a**/**2b**, respectively, upon examination of the crude mixtures).

**Figure 1:**
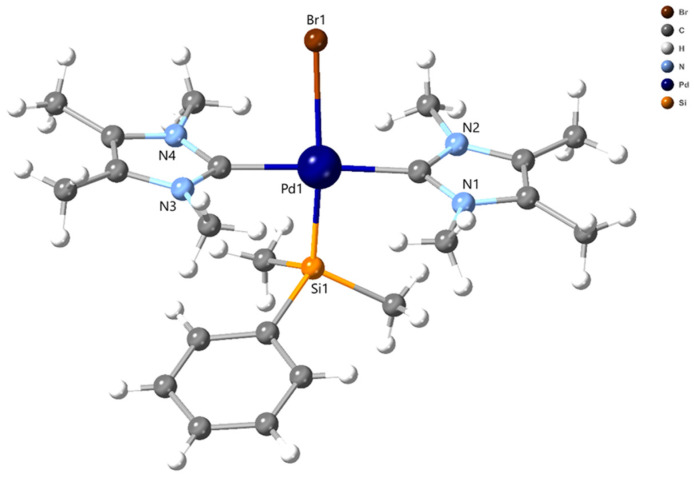
Molecular structure of **2b**. Hydrogen atoms are omitted for clarity. Selected bond lengths [Å] and angles [^o^]: Pd1-Br1 2.6333(7), Pd-Si1 2.2948(18), Pd1-C1 2.028(5), Pd1-C8 2.025(5); C1-Pd1-Br1 94.97(16), C1-Pd1-Si1 89.15(17), C8-Pd1-Br1 87.62(16), C8-Pd1-Si1 88.60(17), C1-Pd1-C8 177.2(2).

In order to further characterize the organometallic complexes, single crystals of **2b** suitable for X-ray analysis were grown by slow evaporation of a saturated deuterated benzene solution at room temperature. X-ray analysis revealed that **2b** displays a marginally distorted square-planar geometry with the two NHCs in a *trans*-configuration and orthogonal to the Br-Pd-Si plane (Figure 1, Table 1).

The carbenic carbon-Pd bond lengths in **2b** [2.028(5) and 2.025(5) Å] are significantly shorter than in *cis*-[Pd(ITMe)_2_(SiMe_2_Ph)_2_] [2.105(3) and 2.123(3) Å], suggesting SiMe_2_Ph exhibits a stronger *trans*-influence than ITMe [15]. The decreased length of the Pd-Si bond in **2b** [2.2948(18) Å] versus *cis*-[Pd(ITMe)_2_(SiMe_2_Ph)_2_] [2.3445(8) and 2.3346(8) Å] infers a stronger Pd-Si bond in **2b** and demonstrates the weak *trans*-influence of Br. Based on these data, the intensity of the *trans*-influence in these two structures follows the sequence: Br < ITMe < SiMe_2_Ph. Thus, the preference for the trans-configuration observed in **2b** may be attributed to the high trans-influence of SiMe_2_Ph and the large steric size of Br.

**Scheme 2:**
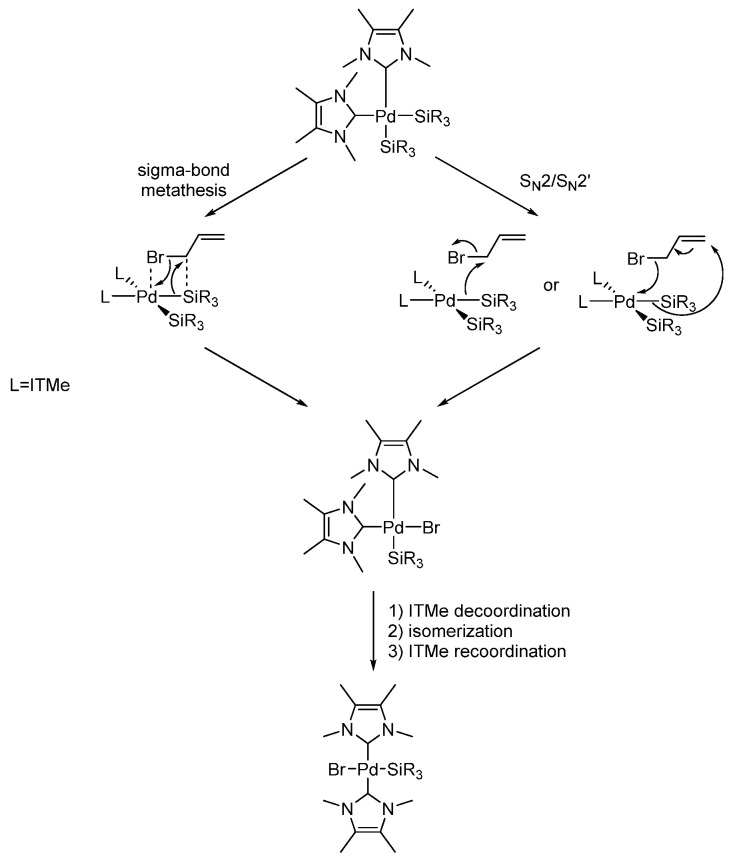
Possible mechanistic routes for the formation of **2**.

A possible mechanism for the formation of **2** includes either a σ-bond metathesis between a Pd-Si, in *cis*-[Pd(ITMe)_2_(SiR_3_)_2_], and Br-C bond, in allylbromide, or an S_N_2/S_N_2′ by the nucleophilic Pd-Si bond at the electrophilic sites in the allyl halide, leading to a *trans* complex. As we have previously suggested using computational studies on related bis-ITMe complexes, an NHC would then dissociate from the palladium center followed by a *cis* to *trans* isomerization of the Br and Si moieties (Scheme 2) [11]. Finally, the dissociated NHC would re-coordinate, constrained by the bulk of the other ligands, in a *cis*-configuration [16,17].

## 3. Experimental

The handling of air-sensitive compounds and their spectroscopic measurements were undertaken using standard Schlenk line techniques using pre-dried Ar (using a BASF R3-11(G) catalyst and 4 Å molecular sieves), or in a MBraun glovebox under N_2_ (O_2_ < 10.0 ppm). All glassware was dried in a 160 °C oven prior to use. Celite was predried in a 200 °C oven and then dried with a heat gun under a dynamic vacuum prior to use. Filter cannulae equipped with microfiber filters were dried in an oven at 160 °C prior to use. Solvents employed in air-sensitive reactions were dried using vacuum distillation, followed by distillation over potassium or stored over activated 4 Å molecular sieves under an Ar atmosphere. NMR spectra were recorded on a Varian VNMRS 400 (Palo Alto, CA, USA) (^1^H 399.5 MHz; ^13^C{1H} 100.5 MHz; ^11^B{1H} 128.2 MHz; ^19^F 375.9 MHz; ^29^Si{^1^H} 79.4 MHz), or 500 (^1^H 499.9 MHz; ^13^C{^1^H} 125.7 MHz). Chemical shifts are reported in ppm. All other experimental details are outlined elsewhere [10].

### Synthesis of trans-[Pd(ITMe)_2_(SiMe_3_)(Br)] *(**2a**)* and Allyltrimethylsilane *(**3a**)*

Allylbromide (0.032 g, 0.26 mmol) was added to a solution of *cis*-[Pd(ITMe)_2_(SiMe_3_)_2_] (0.043 g, 0.09 mmol) in C_6_D_6_ or toluene (3.0 mL) and the resulting reaction mixture was stirred at room temperature for 1.5 h. At this stage, the volatiles were removed in vacuo and the off-white powder was washed with hexane (3 × 4.0 mL).

**2a**, Yield: 0.040 g, 92%. ^1^H NMR (399.5 MHz, C_6_D_6_): δ_H_ = 3.68 [s, 12H, N(1,3)-C**H_3_**], 1.42 [s, 12H, C(4,5)-C**H_3_**], 0.12 [s, 9H, SiMe_3_]. ^13^C{^1^H} NMR (100.5 MHz, C_6_D_6_): δ_C_ = 184.9 [N**C**N], 124.0 [**C**(4,5)-CH_3_], 35.1 [N(1,3)-**C**H_3_], 8.5 [C(4,5)-**C**H_3_], 6.9 [Si**Me_3_**]. ^29^Si{^1^H} NMR (79.4 MHz, C_6_D_6_): δ_Si_ = 7.68. Elem. Anal. Calcd. for C_17_H_33_N_4_SiBrPd: C, 40.20%; H, 6.55%; N, 11.03%. Found: C, 40.15%; H, 6.54%; N, 10.95%. **3a** (from crude reaction solution), ^1^H NMR (399.5 MHz, C_6_D_6_): δ_H_ = 5.77 [m, 1H, C**H**=], 4.92 [m, 1H, C**H**=], 4.89 [m, 1H, C**H**=], 1.44 [m, 2H, C**H_2_**], −0.03 [s, 9H, Si**Me_3_**]. [Agrees with an independently taken ^1^H NMR sample of commercially available allyltrimethylsilane].

### Synthesis of trans-[Pd(ITMe)2(SiMe2Ph)(Br)] *(**2b**)*

Allybromide (6.0 µL, 0.07 mmol) and *cis*-[Pd(ITMe)_2_(SiMe_2_Ph)] (0.021 g, 0.03 mmol) were dissolved in C_6_D_6_ or toluene (1.0 mL). The resulting reaction mixture was stirred at room temperature for 2 h under an N_2_ atmosphere. At this stage, all volatiles were removed in vacuo and the resulting white solid was washed with hexane (3 × 2.0 mL). Yield: 0.018 g, 93%. ^1^H NMR (399.5 MHz, C_6_D_6_): δ_H_ = 7.20 [m, 2H, SiMe_2_**Ph**], 7.07 [m, 3H, SiMe_2_**Ph**], 3.51 [s, 12H, N(1,3)-C**H_3_**], 1.42 [s, 12H, C(4,5)-C**H_3_**], 0.31 [s, 6H, Si**Me_2_**Ph]. ^13^C{^1^H} NMR (100.5 MHz, C_6_D_6_): δ_C_ = 183.4 [N**C**N], 149.6 [SiMe_2_***i*-Ph**], 133.1 [SiMe_2_**Ph**], 127.0 [SiMe_2_**Ph**], 126.5 [SiMe_2_***p*-Ph**], 124.2 [**C**(4,5)-CH_3_], 34.9 [N(1,3)-**C**H_3_], 8.5 [C(4,5)-**C**H_3_], 4.2 [Si**Me_2_**Ph]. ^29^Si{^1^H} NMR (79.4 MHz, C_6_D_6_): δ_Si_ = 2.44. (It was not possible to obtain elemental analysis for **2b**– every attempt resulted in numbers that were inconsistent with calculated values. A possible reason for this is decomposition of **2b** by exposure to air or moisture on transit to data collection).

Crystal data for **2b**: C_22_H_35_N_4_SiBrPd, *M*_r_ = 569.94 g mol^−1^, orthorhombic, space group P2 = 2_1_2_1_, *a* = 10.5467(4) Å, *b* = 14.3455(3) Å, *c* = 16.7301(4) Å, *α* = 90°, *β* = 90°, *γ* = 90°, *V* = 2531.23(13) Å^3^, *Z* = 4, *T* = 173 K, λMo(Kα) = 0.71073, *R*_1_ [*I* > 2*σ*(*I*)] = 0.0345, *wR_2_* (all data) = 0.0677, *GooF* = 1.011.

Crude ^1^H NMR data are consistent with the formation of allyldimethylphenylsilane (**3b**) as a product of this reaction. However, this was not isolated in this instance [18].

## 4. Conclusions

Under mild conditions, non-pincer bis(NHC)(silyl)palladium halide complexes of the type *trans*-[Pd(ITMe)_2_(SiR_3_)(Br)] (SiR_3_ = SiMe_2_Ph (**2a**), and SiMe_3_ (**2b**)) were synthesized, by the reaction of allylbromide with the corresponding complexes *cis*-[Pd(ITMe)_2_(SiR_3_)_2_], **1a** or **1b**, respectively. A possible mechanistic route for the formation of **2** involves either a σ-bond metathesis or an S_N_2/S_N_2′ reaction between allybromide and **1**. This would necessitate a *cis*-*trans* isomerization via dissociation of an NHC ligand-[19]. The reactivity of *trans*-[Pd(ITMe)_2_(SiR_3_)(Br)] is unexplored but will soon be carried out. The facile formation and apparent stability of *trans*-**2** may indeed hinder the catalytic silylation of ally halides mediated by ITMe_2_Pd-based complexes since the adoption of a *cis*-configuration is a prerequisite for reductive elimination and involvement in a catalytic cycle. Solutions to these unexplored questions are currently being sought, e.g., the potential for halide abstraction, and will be reported in due course.

## Figures and Tables

**Table 1:** Crystal data and structure refinement for **2b**.

Empirical formula	C_22_H_35_BrN_4_PdSi
Formula weight	569.94
Temperature/K	173
Crystal system	orthorhombic
Space group	P2_1_2_1_2_1_
a/Å	10.5467(4)
b/Å	14.3455(3)
c/Å	16.7301(4)
α/°	90
β/°	90
γ/°	90
Volume/Å^3^	2531.23(13)
Z	4
ρ_calc_g/cm^3^	1.496
μ/mm^−1^	2.374
F(000)	1160.0
Crystal size/mm^3^	0.22 × 0.2 × 0.15
Radiation	MoKα (λ = 0.71073)
2*θ* range for data collection/°	6.836 to 52.744
Index ranges	−13 ≤ h ≤ 8, −17 ≤ k ≤ 11, −14 ≤ l ≤ 20
Reflections collected	7612
Independent reflections	4799 [R_int_ = 0.0320, R_sigma_ = 0.0568]
Data/restraints/parameters	4799/0/272
Goodness-of-fit on F^2^	1.018
Final R indexes [I >= 2σ (I)]	R_1_ = 0.0322, wR_2_ = 0.0614
Final R indexes [all data]	R_1_ = 0.0376, wR_2_ = 0.0640
Largest diff. peak/hole/e Å^−3^	0.51/−0.34
Flack parameter	0.004(8)
CCDC deposition number	2076437

## Sample Availability

No samples of the compounds are available from the authors.
